# An Overview of Endometriosis and Potential Pharmacogenetic Targets

**DOI:** 10.1007/s11095-026-04049-9

**Published:** 2026-02-25

**Authors:** Noelia Pérez-Gómez, María Ángeles Martínez-Zamora, Francisco Carmona, Jesús Novalbos, Francisco Abad-Santos, Dora Koller, Miriam Saiz-Rodríguez

**Affiliations:** 1 Research Unit, Fundación Burgos Por La Investigación de La Salud (FBIS), Hospital Universitario de Burgos, Burgos, 09006 Spain; 2 Department of Health Sciences, Health Sciences Faculty, University of Burgos, Pº de los Comendadores, s/n, Burgos, 09001 Spain; 3 Department of Gynecology, Clinical Institute of Gynecology, Obstetrics and Neonatology, Hospital Clínic of Barcelona, University of Barcelona, Institut Clínic d´Investigacions Biomèdiques August Pi I Sunyer (IDIBAPS), Barcelona, 08036 Spain; 4 Clinical Pharmacology Department, Hospital Universitario de La Princesa, Universidad Autónoma de Madrid (UAM), Instituto de Investigación Sanitaria La Princesa (IIS-Princesa), Madrid, 28006 Spain; 5 Department of Genetics, Microbiology, and Statistics, Faculty of Biology, University of Barcelona, Barcelona, Catalonia 08028 Spain; 6 Department of Psychiatry, Yale School of Medicine, New Haven, CT 06511 USA

**Keywords:** drug response, endometriosis, pharmacogenetics, precision medicine

## Abstract

**Background:**

Endometriosis is a chronic inflammatory disease characterized by the presence of endometrial-like tissue outside the uterine cavity. It affects approximately 10–15% of women of reproductive age globally and is characterized by heterogeneous symptoms with chronic pelvic pain, dysmenorrhea, and infertility being the most common. Although pharmacological treatments are available to manage its symptoms, many women either do not respond to these therapies or experience adverse drug reactions (ADRs) that outweigh the original symptoms of endometriosis.

**Rationale/Objectives:**

The aim of this review is to provide a comprehensive overview of endometriosis and to identify pharmacogenetic markers that might influence drug response in its treatment and management.

**Outcomes:**

Current research highlights a critical gap in pharmacogenetic biomarkers for endometriosis treatment, limiting the potential for personalized therapeutic strategies.

**Wider Implications:**

Integrative multi-omics approaches combining genetic, inflammatory, and hormonal profiles may enhance patient stratification and optimize individualized care.

## Introduction

Endometriosis is a complex and often debilitating condition that affects approximately 190 million women of reproductive age worldwide [1]. This chronic disease occurs when tissue similar to the uterine lining, known as the endometrium, grows outside the uterus, typically within the pelvic cavity [2]. This misplaced tissue can cause a range of symptoms, including dysmenorrhea, chronic pelvic pain, dyspareunia, infertility, and gastrointestinal issues, among others [2, 3]. Endometriotic lesions can grow on various tissues and organs outside the uterus, and the disease typically manifests in three forms: peritoneal, ovarian, and deep infiltrating endometriosis. Treatment strategies vary significantly based on the extent of the disease and the predominant clinical presentation. [4].

## Aetiology and Pathogenesis of Endometriosis

Despite being a highly prevalent condition, the aetiology and pathogenesis of endometriosis remain poorly understood. The most widely accepted theory suggests that the disease develops due to the retrograde menstruation into the lower abdominal cavity, where it implants and triggers a chronic inflammatory response [4]. However, this mechanism alone does not fully explain the disease, as retrograde menstruation has also been observed in women without endometriosis, suggesting a multifactorial origin. Other theories propose that endometriosis could result from coelomic metaplasia, in which peritoneal cells transform into endometrial-like tissue due to hormonal stimuli or inflammation; or from the dissemination of endometrial fragments through lymphatic and blood vessels, to other abdominal or extra-abdominal locations, where they may form endometriotic lesions [5]. In line with this, the benign metastasis theory suggests that endometrial cells can spread through lymphatic vessels to distant organs, supported by histopathological evidence of endometrial tissue within lymph nodes and by the dysregulation of lymphangiogenic factors such as vascular endothelial growth factors VEGF-C and VEGF-D in affected women [5]. Finally, hormonal, biochemical, immunological, genetic, and environmental factors are also believed to induce the differentiation of the mesothelial epithelium, leading to the formation of endometriotic lesions [6, 7].

Beyond its origins, increasing evidence supports the notion that endometriosis is characterized by persistent inflammation. Elevated levels and concentrations of activated macrophages, interleukin (IL)−1β, IL-6, and IL-8, nerve growth factor (NGF), other immune cells, and inflammatory factors have been detected in the peritoneal fluid, while endometriotic lesions themselves create an inflammatory microenvironment [8]. This environment interacts with endometriotic cells, including stromal and epithelial cells, playing a crucial role in the development and persistence of the disease [8].

## Diagnostic Delay and Impact on Quality of Life

Based on the economic, societal, and physical impacts of endometriosis, it should be considered as a modern epidemic [9]. Endometriosis affects several aspects of life, including participation in daily and social activities, physical and sexual functioning, relationships, educational and work productivity, mental health, and well-being [10]. The potential for the disease to negatively impact the quality of life is substantial, because as an early-onset chronic disease, it follows women from menarche through menopause. However, the average delay in diagnosis is 4–11 years [11]. Traditionally, diagnosis has been established through visual inspection of the pelvis during laparoscopy or laparotomy; however, histological confirmation is not always obtained [12]. Since surgery is invasive and expensive, there has been considerable interest in using minimally invasive techniques, such as imaging, genetic testing, biomarkers, or miRNA tests. However, this remains a challenge, as laparoscopy is still the gold standard and many professionals consider it the only definitive method [12]. ​

## Current Available Medical Treatments of Endometriosis

**Fig. 1:**
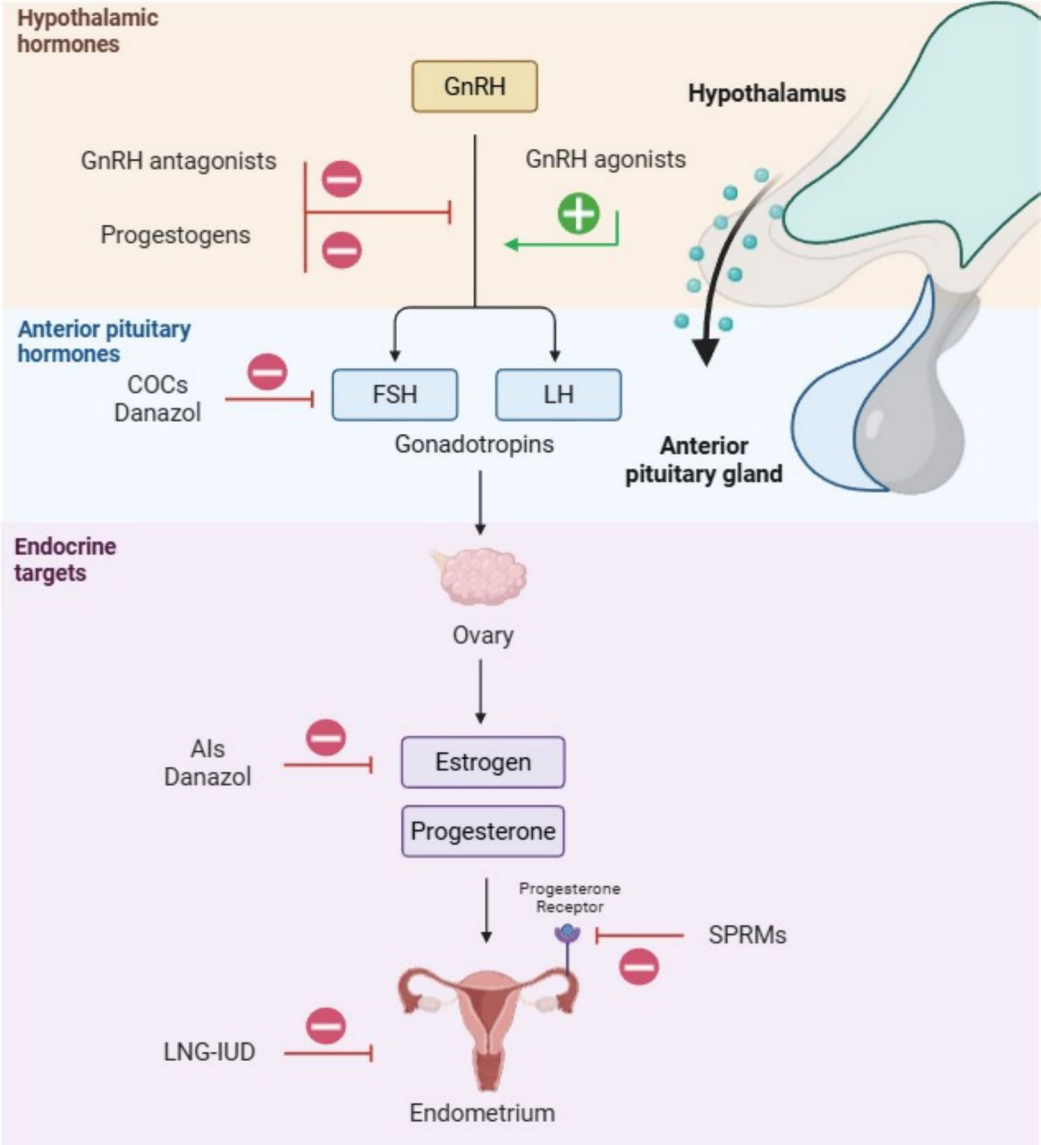
Mechanisms of hormonal modulation in endometriosis. *Abbreviations*: *GnRH* gonadotropin-releasing hormone, *FSH* follicle-stimulating hormone, *LH* luteinizing hormone, *COCs* combined oral contraceptives, *AIs* aromatase inhibitors, *LNG-IUD* levonorgestrel intrauterine device, *SPRMs* selective progesterone receptor modulators.

Endometriosis lacks a permanent cure. The American Society for Reproductive Medicine recommends its lifelong management focusing on optimizing medical treatment and minimizing the need for recurrent surgeries [13]. As per the guidelines provided by the European Society of Human Reproduction and Embryology (ESHRE) in 2022, the most frequently recommended treatments for endometriosis involve medications that modify hormonal levels [14]. Oestrogens play a crucial role in the development of endometriosis by triggering key mechanisms such as macrophage activation, stimulation of major proinflammatory cytokines, and cell proliferation. As a result, therapies aimed at inhibiting their effects have been the standard pharmacological treatment for endometriosis. These medications either suppress ovarian activity or directly target steroid receptors and enzymes found in the lesions. The list of these medications includes progestogens, selective progesterone receptor modulators, combined oral contraceptives, gonadotropin-releasing hormone (GnRH) agonists, GnRH antagonists, levonorgestrel intrauterine devices, danazol, and aromatase inhibitors [14] (Fig. 1). Each of these hormonal treatments results in a notable decrease in pain that is clinically significant when compared to placebo [14]. However, identifying the most effective medication with the fewest adverse drug reactions (ADRs) for each patient still relies on a trial-and-error approach [15]. The currently available therapeutic approaches are presented below:

- *Progestogens*. Progestins are synthetic compounds that decrease the frequency of pulsatile GnRH release, which results in reduced pituitary secretion of follicle-stimulating hormone (FSH) and luteinizing hormone (LH), ultimately leading to the suppression of ovarian steroid production [16]. Available progestins used to treat endometriosis include a variety of oral and depot forms, such as norethisterone acetate, dienogest, desogestrel, cyproterone acetate, depot medroxyprogesterone acetate, levonorgestrel intrauterine device and etonogestrel subdermal implant. These treatments are generally effective in managing pain symptoms in about three out of four women with endometriosis and often provide an affordable alternative treatment option [17]. Nonetheless, their use may cause ADRs, including irregular bleeding, weight gain, mood changes, and breast tenderness [18, 19]. Some patients may also experience nausea, headaches, and decreased libido. The severity and frequency of these ADRs vary depending on the specific progestin used and its route of administration [20].
- *Selective progesterone receptor modulators*. Selective progesterone receptor modulators, including mifepristone, asoprisnil, ulipristal acetate, and telapristone acetate, are a class of progesterone receptor ligands that exhibit both agonistic and antagonistic properties. In the absence of progesterone, they act as weak progestins, while in its presence, they may exert mild antiprogestogenic effects, particularly in the endometrium. These characteristics render them potential therapeutic agents for conditions such as uterine fibroids and endometriosis [21]. In patients with uterine fibroids, mifepristone and ulipristal acetate have consistently demonstrated clinical efficacy, while studies of asoprisnil and telapristone were discontinued due to safety-related concerns. Mifepristone has also demonstrated utility for the management of endometriosis. However, evidence supporting the efficacy of asoprisnil, ulipristal acetate, and telapristone acetate for this condition remains limited [22]. One of the notable ADRs of selective progesterone receptor modulators is related to their hypoestrogenic effects, which could potentially lead to symptoms such as vasomotor instability, similar to menopausal symptoms; these may include hot flashes, night sweats, and osteoporosis over long-term usage [23]. Additionally, selective progesterone receptor modulators are often linked to gastrointestinal disturbances like nausea and abdominal pain, as well as changes in mood or emotional state. The anti-ovulatory effect of these medications also implies contraceptive effects, which may be either desirable or undesirable depending on patient’s reproductive plans [24].
- *Combined oral contraceptives*. Oral contraceptives contain synthetic oestrogens and progestins that exert negative feedback on the hypothalamic-pituitary–gonadal axis, suppressing the release of GnRH, FSH, and LH, thereby reducing endogenous estrogen production and inhibiting ovulation. By suppressing ovarian activity, they may also decrease prostaglandin production derived from endogenous oestrogens, thus reducing inflammation. Due to their better tolerability and lower metabolic impact compared to GnRH agonists and danazol, combined oral contraceptives — whose continuous use has shown greater efficacy than cyclic administration — are considered the first-line treatment for endometriosis, either as an alternative to surgery or post-surgical support [25]. However, the use of combined oral contraceptives frequently causes ADRs, including nausea, irregular bleeding or menorrhagia, and spotting, with the latter being commonly intensified in continuous regimens and challenging to manage in some patients [26]. Cardiovascular complications, although reduced with lower estrogen and progestin dosages, remain a significant concern, and these ADRs are the primary reason for discontinuation [27].
- *GnRH agonists*. GnRH agonists, modified versions of native GnRH, reduce receptor sensitivity in the pituitary gland, leading to a hypogonadotropic state that suppresses endometriosis and prevents new lesions. These drugs, including goserelin, leuprolide, and nafarelin, effectively relieve pain and shrink endometriomas. Although as effective as combined oral contraceptives and other hormonal therapies, these agents are considered second-line treatments when first-line options fail or are not tolerated. Their use is limited by a high relapse rate (approximately 50% of women experience recurrence within six months) and by the induction of a transient pharmacologic menopause, which is associated with significant ADRs, including vasomotor instability, vaginal dryness, headaches, bone demineralization, cholesterol imbalance, depression, hot flashes, and reduced libido [25, 28].
- *GnRH antagonists*. GnRH antagonists, including elagolix, relugolix, and linzagolix, bind to pituitary GnRH receptors and rapidly suppress gonadotropin and gonadal steroid production, providing effective non-invasive management of endometriosis-related pain [25, 29, 30]. While generally well-tolerated, their use can induce a hypoestrogenic state, leading to hot flushes, night sweats, vaginal dryness, decreased libido, and some degree of bone mineral density loss if used without add-back therapy [29, 31]. However, add-back therapy with low-dose estrogen and/or progestin mitigates these effects, particularly in patients receiving relugolix or linzagolix, enhancing long-term safety and tolerability [29, 30].
- *Levonorgestrel intrauterine device*. Levonorgestrel intrauterine device releases levonorgestrel, a potent 19-nortestosterone derivative, into the uterine cavity at a steady rate of 20 μg/day over a 5-year period. It significantly affects the eutopic endometrium, causing it to become atrophic and inactive, while ovulation is typically not inhibited. Levonorgestrel intrauterine system aids in the treatment of endometriosis by inducing atrophy of endometrial glands, promoting decidual transformation of the stroma, reducing endometrial cell proliferation, and increasing apoptosis. Additionally, its beneficial effects on endometriosis symptoms are likely linked to a decrease in the expression of estrogen and progesterone receptors in the ectopic endometrium, as well as an increase in Fas (a pro-apoptotic receptor, that promotes programmed cell death in endometrial cells) expression in both eutopic and ectopic endometrial tissue [17]. Regarding ADRs, in the initial months of treatment, menstrual irregularities such as spotting and menorrhagia are common. After a year of use, approximately 20–30% of patients experience amenorrhea. Other common ADRs include nausea, headache, breast tenderness, mood changes, and acne [32]. Less common ADRs identified include anorexia, ectopic pregnancy, exanthema, chloasma, miscarriage, and weight gain [32]. Therefore, the best candidates for this treatment modality are women who do not tolerate progestins used systemically as well as those whose personal circumstances favour a long-term treatment option that does not require frequent changes in therapeutic strategy [17].
- *Danazol*. The synthetic androgen 2,3-isoxazol (danazol), a derivative of 17α-ethynyl testosterone, exhibits mild androgenic effects and strong antiestrogenic activity. It works by inhibiting gonadotropin release, competitively blocking steroidogenic enzymes, modulating immune function, and suppressing cell proliferation. Numerous studies have shown that danazol is effective in reducing pain symptoms associated with endometriosis [25]. However, its use may be limited by androgenic ADRs, such as weight gain, acne, hirsutism, elevated transaminases, mood changes, hot flashes or interference on the regularity of menstrual cycles [25]. These ADRs tend to peak around four months of treatment and then decrease in severity. Interestingly, the route of administration can significantly impact the ADR profile. While oral danazol therapy is associated with androgenic and metabolic ADRs, vaginal administration of danazol may minimize these systemic ADRs [33]. Nevertheless, the ESHRE in their latest guideline recommends that danazol should only be considered when no other medical therapy is available due to its unfavourable ADR profile [14].
- *Aromatase inhibitors*. By inhibiting aromatase activity, aromatase inhibitors impair the conversion of androgens into oestrogens. In addition, similarly to danazol, they suppress ovarian and local estrogen formation in endometriotic tissue [25]. The third-generation aromatase inhibitors (anastrozole, letrozole, and exemestan) are more effective than the original aminoglutethimide and have greater specificity for the aromatase enzyme, but they are only approved for the treatment of breast cancer. Moreover, ADRs associated with them, such as headache, nausea, and diarrhoea are less common [25]. However, the ESHRE in their latest guideline recommends the use of aromatase inhibitors associated to another hormonal treatment (combined oral contraceptives, progestogens, or GnRH analogues) only in women in whom all surgical and medical treatments have failed, given the limited evidence supporting their efficacy and the potential for significant hypoestrogenic ADRs such as bone loss and vasomotor symptoms [14, 21].
- *Non-steroidal anti-inflammatory drugs (NSAIDs)*: NSAIDs are widely used for pain management in endometriosis, due to their efficacy in reducing inflammation and providing analgesic effects. However, these drugs are responsible for 21–25% of reported ADRs, including both immunological and nonimmunological hypersensitivity reactions [34]. In addition, they are associated with gastrointestinal toxicity, such as gastric ulcers and other serious complications, as well as an increased risk of cardiovascular ADRs [35].

Given the trial-and-error paradigm, pharmacogenetic approaches may offer a way to predict individual responses. In the next section, we review potential pharmacogenetic targets for endometriosis therapies.

## Search Methodology

For the preparation of this narrative review, a non-systematic but comprehensive search was conducted in PubMed/MEDLINE, Scopus and Web of Science, complemented by manual screening of reference lists from key articles and international clinical guidelines (ESHRE, NICE, ASRM), with no initial restriction on study design, focusing on the pathophysiology, diagnosis and medical management of endometriosis. The main search period covered 2000 to 2026, while also incorporating earlier classical works when their conceptual or historical relevance was necessary to contextualize specific pathophysiological mechanisms. Keyword combinations such as “endometriosis,” “pathogenesis,” “diagnosis,” “management,” “oral contraceptives,” “progestins,” “GnRH antagonists,” “pharmacogenetics,” “drug metabolism,” “inflammation,” “oxidative stress,” and equivalent MeSH terms were used. Although several recent high-quality studies were identified, evidence gaps persist in some areas—particularly in molecular pathophysiology, pharmacogenomics, and ADRs of hormonal treatments—which necessitated the citation of older studies where more recent data were unavailable.

## Potential Pharmacogenetic Targets and Inflammatory Biomarkers

Given the treatments outlined above, we now explore how patient-specific genetic and molecular factors might influence the response to those treatments. Pharmacogenetics is the field that investigates how genetic variations impact drug metabolism, efficacy, and potential ADRs. By unravelling these genetic factors, healthcare providers may tailor medication selection and dosing to optimize treatment outcomes and reduce the risk of ADRs for each patient. Pharmacogenetics offers the potential for personalized medicine, where treatment approaches are customized based on an individual's genetic characteristics, leading to more effective and safer therapeutic interventions.

Despite the high prevalence and significant impact of endometriosis on patients’ quality of life, there is a lack of comprehensive studies examining the pharmacogenetic aspects of various treatment options, including hormonal therapies, pain medications, and emerging targeted therapies. Some potential pharmacogenetic biomarkers that may influence response to endometriosis treatment include enzymes involved in drug metabolism (e.g. CYP3A4/5, CYP2C9, CYP2D6), drug transporters (e.g. ABCB1) and drug target receptors (e.g. ESR1/2, PGR) (Table I); regulatory genes (Table II), as well as inflammatory markers associated with endometriosis pathophysiology (e.g. IL6, TNF, MMP9) (Table III).

**Table I:** Potential Pharmacogenetic Targets in Endometriosis Treatment

Drug	Biomarkers	Clinical effect	References
Ethinylestradiol, Progestogens (drospirenone, levonorgestrel, dienogest, desogestrel, norethindrone, progesterone)	*CYP3A4* (rs2740574, rs35599367); *CYP3A5 (*rs776746*)*; *CYP2C9 (*rs1799853*,* rs1057910*)*; *CYP2C19* (rs4244285, rs4986893, rs12248560); *ESR1* (rs2234693, rs9340799);*ESR2* (rs4986938, rs1256049); *PGR* (rs1042838, rs10895068)	CYP variants alter metabolism → ↓ efficacy ↑ ADRs; receptor polymorphisms linked to progesterone resistance, infertility, variable therapeutic response	[36–45]
GnRH antagonists (elagolix, relugolix)	*CYP3A4* (rs2740574, rs35599367); *CYP3A5 (*rs776746*)*; CYP2C8 (rs11572080, rs10509681, rs1058930); *ABCB1* (rs1045642, rs2032582, rs1128503)	Variability in drug exposure, ovarian suppression, and hypoestrogenic ADRs	[46–50]
Aromatase inhibitors (anastrozole, letrozole)	*CYP19A1* (rs4646, rs10046, rs700518, rs700519)	Variability in oestrogen suppression, treatment response, and ADRs (bone loss, menopausal symptoms)	[51–53]
NSAIDs (ibuprofen, naproxen, diclofenac, ketoprofen, dexketoprofen)	*CYP2C9* (rs1799853, rs1057910, rs28371685); *ABCB1* (rs1045642, rs2032582, rs1128503); *PTGS1* (rs10306114, rs3842787, rs5789); *PTGS2* (rs20417, rs689465, rs5277, rs5275)	Altered metabolism and efficacy. CYP2C9 PM ~ 30–80% reduction in drug clearance and a ~ 2–threefold increase in systemic exposure, associated with increased ADR risk (gastrointestinal, renal, cardiovascular). *ABCB1* alleles linked to lower plasma levels (dexketoprofen)	[54–59]
Opioids (tramadol, codeine)	CYP2D6 phenotypes (PM, UM); *OPRM1* A118G (rs1799971)	CYP2D6 PM → reduced metabolite formation and inadequate analgesic response; CYP2D6 UM → > twofold increased exposure to active metabolites, leading to increased risk of oversedation/respiratory depression. OPRM1 variant affects opioid binding, pain perception, and response	[60–65]

*CYP* cytochrome P450, *ESR* oestrogen receptor, *PGR* progesterone receptor, *ADRs* adverse drug reactions, *GnRH* gonadotropin-releasing hormone, *ABCB1* ATP binding cassette subfamily B member 1, *NSAIDs* non-steroidal anti-inflammatory drugs, *PTGS* prostaglandin-endoperoxide synthase, *OPRM* opioid receptor mu 1, *PM* poor metabolizer, *UM* ultra-rapid metabolizer

**Table II:** Potential Regulatory Biomarkers Influencing Hormonal Therapy Response in Endometriosis

Regulatory factor	Gene/Variant	Clinical effect	References
PXR	*NR1I2* polymorphisms (Q158K)	Modulates CYP3A4 expression → alters metabolism of combined oral contraceptives, progestins, GnRH antagonists	[66–69]
SHBG	*SHBG* (rs1799941, rs6257)	Alters SHBG levels → modifies free estrogen/androgen bioavailability and hormonal therapy response	[70, 71]

*PXR *pregnane X receptor, *NR1I2* nuclear receptor subfamily 1 Group I Member 2, *CYP* cytochrome P450, *GnRH* gonadotropin-releasing hormone, *SHBG* sex hormone-binding globulin

**Table III:** Potential Inflammatory and Tissue Remodelling Biomarkers in Endometriosis

Molecular pathway	Biomarkers	Clinical effect	References
Cytokines	IL-6, IL-1β, TNF-α, IL-8, IFN-γ	Elevated levels correlate with pain, inflammation, and lesion progression; potential predictors of treatment response. Hormonal therapies (progestins, combined oral contraceptives, dienogest) suppress IL-6, TNF-α, IL-1β, and IL-8 via PGR–mediated nuclear factor kappa B inhibition	[72–84]
Oxidative stress	MPO, SOD	MPO ↑, SOD ↓ in endometriosis; oxidative stress correlates with disease severity. Hormonal contraceptives normalize oxidative balance. Antioxidant therapy (N-acetylcysteine, curcumin, vitamins C/E) enhances SOD activity and reduces oxidative damage → improving symptoms and fertility	[85–90]
ECM remodeling	MMP-9, TIMP-1 imbalance	↑ MMP-9/TIMP-1 ratio → ECM breakdown, lesion invasion, fibrosis progression. Hormonal therapies (progestins, dienogest) modulate MMPs and PGR expression; resveratrol and other natural compounds reduce MMP expression	[58, 80, 91–98]

*IL* interleukin, *TNF* tumour necrosis factor, *IFN* interferon, *PGR* progesterone receptor, *MPO* myeloperoxidase, *SOD* superoxide dismutase, *ECM* extracellular matrix, *MMP* matrix metalloproteinase, *TIMP* tissue inhibitor of metalloproteinases

### Enzymes Involved in Drug Metabolism

The cytochrome P450 (CYP) superfamily consists of membrane-bound haemoproteins that play a central role in drug detoxification, cellular metabolism, and homeostasis. Among them, isoforms from the CYP1, CYP2, and CYP3 families account for the metabolism of nearly 80% of drugs in clinical use [99]. Because of this, first-line hormonal therapies for endometriosis — such as combined oral contraceptives, progestogens, GnRH agonists/antagonists, and selective hormonal modulators — are significantly affected by CYP-mediated metabolism, which directly influences their pharmacokinetic profile.

Ethinylestradiol and progestogens, including drospirenone, levonorgestrel, dienogest, desogestrel, and norethindrone, are primarily metabolized by CYP3A4 and CYP3A5. While specific single nucleotide polymorphisms (SNPs) have not been evaluated yet, general insights about these enzymes can be gathered from other medication groups. For instance, CYP3A4*1B (rs2740574), CYP3A4*22 (rs35599367), and CYP3A5*3 (rs776746) variants [36], have been shown to alter enzyme activity and expression, leading to interindividual variability in drug metabolism, as observed for tacrolimus. When these variants are combined with exposure to strong CYP3A inducers (e.g. rifampicin, carbamazepine), there can be a significant reduction in drug efficacy and an increase in breakthrough bleeding [37]. Additionally, *CYP2C9* polymorphisms, notably CYP2C9*2 (rs1799853) and CYP2C9*3 (rs1057910), have been shown to influence the metabolism of norethindrone, desogestrel, and progesterone, key components of hormonal contraceptive therapy, potentially modifying contraceptive efficacy and ADRs [38]. Variants in *CYP2C19* may also contribute to variability in progesterone disposition. In particular, CYP2C19*2 (rs4244285) and CYP2C19*3 (rs4986893) are associated with increased progesterone exposure, whereas CYP2C19*17 (rs12248560) is linked to enhanced metabolism and reduced plasma levels [39].

The GnRH antagonists elagolix and relugolix undergo metabolism via CYP3A4, and in the case of relugolix also via CYP3A5 [46, 47]. Interindividual variability in these pathways may alter drug exposure and contribute to differences in ovarian suppression and hypoestrogenic ADRs. Additionally, while relugolix is partially metabolized by CYP2C8 [46], no pharmacogenetic studies have yet assessed the impact of *CYP2C8* variants on its pharmacokinetics or efficacy. However, variants such as CYP2C8*3 (rs11572080, rs10509681), and CYP2C8*4 (rs1058930) have been shown to alter the metabolism and systemic exposure of other CYP2C8 substrates, including paclitaxel and repaglinide [48–50]. While no endometriosis-specific data exist, these variants may plausibly alter GnRH antagonist exposure by analogy to other drugs. Lastly, according to the European Medicines Agency (EMA) assessment report, linzagolix is not significantly metabolized by a single CYP pathway [100]. Hence, further research is needed to better characterize its metabolic pathways and to determine whether genetic variation may influence its pharmacokinetics or clinical efficacy.

CYP19A1 catalyses androgen-to-estrogen conversion and represents the direct target of aromatase inhibitors (anastrozole, letrozole) [51]. Although no studies have specifically investigated the impact of *CYP19A1* variants in women with endometriosis treated with aromatase inhibitors, evidence from patients with breast cancer indicates that certain polymorphisms in this gene are associated with variability in treatment response and with ADRs, including bone loss and menopausal symptoms [52, 53].

The Clinical Pharmacogenetics Implementation Consortium (CPIC) guidelines highlight that polymorphisms in the *CYP2C9* gene significantly impact the metabolism of NSAIDs [54], such as ibuprofen, naproxen, and diclofenac, which are commonly prescribed for pain management in endometriosis patients. CYP2C9*2 (rs1799853) and CYP2C9*3 (rs1057910) variants, can significantly alter drug metabolism, increasing susceptibility to ADRs like renal, cardiovascular and hepatotoxicity, as well as gastrointestinal bleeding [54–56]. Moreover, the structural changes induced by *CYP2C9* SNPs rs1057910 (CYP2C9*3) and rs28371685 (CYP2C9*11) alter the enzyme’s active site, impairing its catalytic efficiency and contributing to ADRs [57]. Finally, the CYP2D6 enzyme metabolism plays a role in the efficacy and safety of tramadol and codeine, strong pain relievers for severe endometriosis cases. CYP2D6 poor metabolizers (PM) may experience lack of analgesic efficacy, whereas ultra-rapid metabolizers (UM) may experience increased production of active metabolites, potentially leading to oversedation and respiratory depression. A study found that women were more likely to report ADRs to tramadol compared to men, particularly gastrointestinal, skin and nervous system issues [60]. This genetic factor seems to have a stronger influence in women, with female normal and intermediate metabolizers experiencing more ADRs than men [60]. The CPIC guidelines strongly recommend genotype-guided prescribing for codeine and tramadol [61].

### Drug Transporters

Drug transporters influence absorption, tissue distribution, and clearance of several endometriosis-related treatments. The ATP binding cassette subfamily B member 1 (*ABCB1*) gene encodes P-glycoprotein, a key efflux transporter [101]. Polymorphisms such as rs1045642 and rs2032582 have been associated with altered pharmacokinetics and therapeutic response in other drug classes, including antiepileptics [102], suggesting that these variants may contribute to interindividual variability in drug response. In the context of endometriosis, *ABCB1* variants can influence the pharmacokinetics of the GnRH antagonists elagolix and relugolix [46, 47]. Conversely, linzagolix is not regarded as a sensitive substrate for major drug transporters, according to the EMA assessment report [100]. *ABCB1* polymorphisms have also been shown to affect the disposition of NSAIDs. In particular, Mejía-Abril *et al*. reported that the C3435TT and G2677A/TA/T alleles were associated with lower plasma concentrations of dexketoprofen [58], indicating that these variants may alter NSAID absorption and distribution.

### Drug Target Receptors

Receptor genes also modulate hormonal treatment sensitivity. Variants in *ESR1* and *ESR2* (oestrogen receptors) and in *PGR* (progesterone receptor) have been associated with functional differences in estrogenic/progestogenic signalling and with progesterone resistance, which may explain why some patients show poor clinical response to dienogest or other progestins [40–42]. In particular, rs2234693 and rs9340799 polymorphisms in *ESR1* have been investigated in several populations, with associations reported between these variants and endometriosis risk or infertility [43, 44]. Similarly, the rs4986938 and rs1256049 polymorphisms in *ESR2* have been linked to endometriosis susceptibility and idiopathic thin endometrium, suggesting functional effects of estrogen receptor β variants [44, 45]. Regarding *PGR*, rs1042838 and rs10895068 variants, have been associated with endometriosis risk and endometrial receptivity disorders, highlighting the potential role of progesterone receptor polymorphisms in clinical variability and progesterone resistance [44, 45]. Furthermore, variants in *PTGS1* and *PTGS2* (Prostaglandin-Endoperoxide Synthase 1 and 2), encoding COX-1 (Cyclooxygenase-1) and COX-2 (Cyclooxygenase-2), have been associated with altered NSAIDs efficacy and ADR profiles in patients with endometriosis, suggesting that genetic variability in these genes contributes to individual differences in response to anti-inflammatory therapy [59]. Additionally, the *OPRM1* (Opioid Receptor Mu 1) A118G polymorphism modulates mu-opioid receptor binding and clinical response, adding a further layer of variability in analgesic efficacy [62, 63]. Evidence from studies in women with primary dysmenorrhea and other pain-related gynecological conditions indicates that carriers of the G allele may have altered pain processing and reduced response to opioid analgesics, suggesting potential implications for personalized pain management in endometriosis [64, 65].

### Regulatory Genes

Regulatory genes such as *NR1I2* (Pregnane X Receptor, PXR) play a central role in modulating CYP3A4 expression, thereby influencing the metabolism of hormonal contraceptives and GnRH antagonists. PXR acts as a xenobiotic sensor that regulates the transcription of CYP3A4 and other drug-metabolizing enzymes, and its activity can be affected by genetic polymorphisms as well as exposure to PXR inducers, leading to interindividual differences in drug efficacy and safety [66–68]. A study identified a novel SNP, Q158K in the *PXR* gene among Chinese subjects. This variant significantly reduced CYP3A4 promoter activity in response to certain PXR ligands. This finding underscores the potential variability in drug metabolism due to *PXR* polymorphisms, directly affecting how individuals metabolize hormonal contraceptives and GnRH antagonists, which are substrates of CYP3A4 [69].

Similarly, sex hormone-binding globulin (SHBG) regulates the proportion of free *versus* bound sex steroids, which is critical for hormonal bioavailability and therapeutic response. Variations in SHBG levels, influenced by both genetic factors and hormonal interventions, can modify the activity of oestrogens and androgens; for instance, elevated SHBG induced by estrogen-containing contraceptives reduces the fraction of free testosterone, whereas danazol decreases SHBG, increasing free androgen levels [70, 71]. Specific SNPs in the *SHBG* gene, such as rs1799941 (A/G) and rs6257 (T/C), have been investigated for their potential association with altered SHBG levels. Some studies have suggested that the G allele of rs1799941 and the T allele of rs6257 may be linked to lower SHBG concentrations [70], but these findings are not universally consistent across different populations. The interplay between regulatory genes like *NR1I2* and *SHBG*, along with environmental and hormonal influences, determines steroid hormone availability and activity (Table II), highlighting the importance of pharmacogenetic profiling for optimizing hormonal therapy efficacy and safety in patients.

### Inflammatory, Oxidative Stress and Matrix Remodelling markers

Multiple molecular pathways contribute to the complex pathophysiology of endometriosis. Among them, inflammatory signalling, oxidative stress, and matrix remodelling have emerged as key processes driving lesion establishment, progression, and associated symptoms (Table III). These mechanisms not only shape the local disease environment but may also influence therapeutic response, representing a comprehensive approach to personalized medicine in the treatment of endometriosis.

#### Cytokines and Inflammatory Mediators

Cytokine dysregulation is a hallmark of endometriosis. Elevated levels of IL-6, IL-10, IL-1β, and tumour necrosis factor-alpha (TNF-α) have been consistently reported in serum and peritoneal fluid of patients, correlating with disease stage, severity and, in some studies, pain manifestations [72–74]. IL-6 in particular has been associated with the degree of lesion proliferation and angiogenesis, and is considered a promising biomarker of disease activity [75]. TNF-α plays a central role by enhancing vascular permeability, stimulating the release of other pro-inflammatory mediators, and promoting angiogenesis within ectopic lesions, thereby sustaining the chronic inflammatory milieu [73, 74]. In addition, IL-8 (CXCL8) and interferon-gamma (IFN-γ) contribute to immune regulation, inflammation, and tissue remodelling in eutopic and ectopic endometrium [73].

Hormonal therapies indirectly modulate these inflammatory pathways, providing a mechanistic basis for their therapeutic efficacy. Progestins and combined oral contraceptives suppress IL-6, TNF-α, and IL-1β production in endometriotic tissue, with dienogest demonstrating particularly robust anti-inflammatory effects through downregulation of IL-6, IL-8, and Monocyte Chemoattractant Protein-1 (MCP-1) expression via both PGR isoforms A and B [76–78]. These anti-inflammatory actions occur through PGR-mediated inhibition of nuclear factor kappa B activation, a key transcription factor driving inflammatory cytokine production in endometriotic cells [79]. Although hormonal therapy reduces systemic inflammatory markers, IL-6 levels may remain elevated, suggesting incomplete resolution of inflammation [80]. This variable suppression of inflammatory markers may explain why 25 to 34% of patients experience pain recurrence within 12 months of discontinuing hormonal therapy [81].

Given their central role in disease pathogenesis, targeting these mediators has emerged as a potential therapeutic strategy. However, despite their indirect anti-inflammatory effects, the abovementioned therapies are not specifically approved for targeted cytokine regulation in endometriosis. To date, only indirect approaches such as antidepressants that suppress TNF-α and increase IL-10, or biological agents used in autoimmune diseases (e.g., TNF-α blockers used in arthritis), may modulate inflammatory pathways, suggesting that similar strategies could be explored in individualized endometriosis management [82–84].

#### Oxidative Stress and Immune Response

Oxidative stress is a significant factor in the pathogenesis and progression of endometriosis. This stress arises from an imbalance between pro-oxidants and antioxidants, where markers like myeloperoxidase (MPO) and superoxide dismutase (SOD) are crucial indicators. MPO, an enzyme found in neutrophils, generates reactive oxygen species that contribute to oxidative stress, while SOD is an antioxidant that mitigates oxidative damage by catalysing the dismutation of superoxide radicals into less harmful molecules [85]. In endometriosis, the presence of oxidative stress is marked by elevated levels of MPO and decreased activity of SOD, suggesting that oxidative damage plays a role in disease progression. Higher oxidative stress is associated with severe stages of endometriosis, highlighting the need for interventions that can manage oxidative stress in affected individuals [86, 87].

Therapeutic management of oxidative stress in endometriosis can involve both hormonal and antioxidant approaches.. Hormonal contraceptives significantly improve oxidative stress balance in women with endometriosis, particularly those with deep infiltrating disease, by increasing antioxidant defences (free oxidant radical defence) and reducing the free oxygen radical/free oxidant radical defence ratio to levels comparable with healthy controls [88]. This improvement in oxidative homeostasis may contribute to the clinical efficacy of hormonal suppression. Notably, while hormonal therapy increases both oxidative stress markers and antioxidant defences in healthy women (maintaining a stable balance), in women with endometriosis it selectively enhances antioxidant capacity without increasing oxidative stress, thereby normalizing the redox balance [88]. Antioxidant supplementation may provide additive benefits, particularly in patients with incomplete response to hormonal therapy. Agents such as N-acetylcysteine, curcumin, and vitamins C and E have been investigated for their potential to reduce oxidative stress and alleviate symptoms of endometriosis. These antioxidants can enhance the body's defence against oxidative damage by replenishing antioxidant enzyme activities such as SOD and mitigating the effects of free radicals [89]. Furthermore, studies indicate that targeting intracellular pathways influenced by oxidative stress could offer therapeutic benefits. For instance, the activation of the nuclear factor erythroid 2–related factor 2 (Nrf2) antioxidant pathway, which enhances the expression of antioxidant enzymes like SOD, presents a promising approach to managing oxidative damage in endometriosis [90]. This complementary strategy could be combined with hormonal suppression in personalized treatment regimens, particularly for patients with persistent oxidative stress despite hormonal therapy.

#### Matrix Remodelling and Fibrosis

Matrix remodelling processes further contribute to lesion invasion. Matrix Metalloproteinase-9 (MMP-9) helps break down and reshape the extracellular matrix (ECM), which allows endometrial tissue to implant in the wrong places. Studies show that women with endometriosis have more MMP-9 in their normal endometrial tissue compared to women without endometriosis [91, 92]. This extra MMP-9 increases the breakdown activity in these tissues, facilitating their growth in ectopic locations. Tissue Inhibitor of Metalloproteinases-1 (TIMP-1) naturally stops MMPs, like MMP-9, and helps keep the ECM stable by controlling MMP activity. In women with endometriosis, there is an imbalance between MMP-9 and TIMP-1, which contributes to the disease. The ratio of MMP-9 to TIMP-1 is much higher in endometriosis, suggesting an imbalance that leads to ECM breakdown and tissue invasion, key features of endometriosis [93].

Hormonal therapies differentially modulate matrix remodelling pathways, with important implications for treatment selection. Progestins inhibit MMP expression through PGR signalling, though this represents one of the molecular targets that may resist progestin action in some endometriotic cells due to progesterone resistance [40]. Dienogest uniquely increases PGR isoform B expression, potentially overcoming this resistance and enhancing MMP suppression [94]. Recent evidence demonstrates that hormonal treatment reduces fibrosis in deep infiltrating endometriosis by decreasing collagen type I and TIMP-1 expression, though it has minimal effect on ovarian endometriomas, highlighting phenotype-specific responses [80, 95]. However, fibrosis may persist despite hormonal therapy, as elevated IL-6 and fibrotic changes in ectopic endometrium are not fully reversed by hormonal suppression, suggesting that combination approaches targeting both hormonal and inflammatory-fibrotic pathways may be necessary for optimal outcomes [80].

Understanding these processes may help finding treatments that focus on restoring the balance between MMP-9 and TIMP-1, reducing the invasive and fibrotic traits of endometriosis. Preclinical studies have investigated the use of MMP inhibitors such as batimastat and marimastat, which have shown efficacy in suppressing matrix degradation and tumour invasion in experimental models. However, their clinical application has been severely limited due to poor pharmacokinetic properties and ADRs, particularly musculoskeletal toxicity observed in patients during clinical trials [96]. Alternatively, approaches using natural plant metabolites, such as flavonoids, terpenoids, and phenolic compounds, have shown potential to modulate apoptotic pathways and suppress ectopic lesion growth in endometriosis. [97]. Among these, resveratrol, a natural compound found in grape skins and red wine, has shown potential in reducing the expression of MMP-2 and MMP-9 in women with endometriosis. A study indicated that resveratrol administration led to a decrease in both mRNA and protein levels of these MMPs in the endometrium, suggesting its role in moderating the inflammatory processes involved in endometriosis [98]. Such findings offer promising avenues for treatment. However, more clinical studies are necessary to establish effective, long-term therapeutic strategies tailored to individual patient needs.

## Limitations of Current Evidence and Future Perspectives

Despite extensive candidate-gene research, reproducibility of associations remains a challenge. Many studies are constrained by small sample sizes, limited ethnic diversity, and a lack of replication, which undermines the generalizability and robustness of their findings. Moreover, the effect of SNPs is often modest, highlighting the need for polygenic and integrative approaches. Analysing several polymorphisms combined with environmental exposures and blood biomarkers could facilitate patient stratification [103, 104]. For instance, one could hypothesize that elevated IL-6 with CYP3A4 PM profile may identify individuals at risk of reduced progestin efficacy, while carriers of *OPRM1* G alleles with high MPO levels may predict poor opioid response in inflammatory pain states. Multi-omics strategies that combine genotyping, transcriptomics, proteomics, and biomarker quantification hold promise for refining personalized management of endometriosis and overcoming the limitations of single-gene studies. For instance, single-cell and spatially resolved multi-omics approaches have revealed distinct transcriptional and metabolic signatures in ovarian endometriomas, identifying novel molecular targets and pathways relevant to disease progression and therapeutic resistance [105, 106]. Integrated metabolomic and proteomic analyses have also improved diagnostic accuracy compared to single-omics assays, supporting the clinical utility of multi-omics panels for non-invasive detection [107]. However, these approaches are still largely in the discovery phase and not yet routine in clinical practice. Future studies integrating multi-omics data with pharmacogenetic and clinical information are essential to translate these insights into personalized therapeutic strategies for endometriosis patients.

## Conclusions

Ongoing research investigates various biomarkers for endometriosis diagnosis and monitoring, but no study specifically addresses pharmacogenetic biomarkers for treatment adjustment. Our review identified numerous candidate genes (e.g., *CYP3A* variants, *ABCB1*, *ESR1/2* polymorphisms), alongside inflammatory, oxidative, and matrix remodelling biomarkers, that influence the metabolism or action of endometriosis therapies, but none have yet been translated into clinical practice. This gap hinders personalized treatment development, potentially leading to suboptimal management strategies. Integration of molecular biomarkers with pharmacogenetic predictors of treatment response represents the foundation for precision medicine in endometriosis management, thereby enabling more agile and accurate treatment adjustments or the rational combination of therapies to enhance efficacy while minimizing adverse effects. Such studies could pave the way for tailored therapeutic interventions, improving patient care and quality of life for those affected by this condition.
